# Neuropsychological mechanisms of falls in older adults

**DOI:** 10.3389/fnagi.2014.00064

**Published:** 2014-04-09

**Authors:** Yu Liu, John S. Y. Chan, Jin H. Yan

**Affiliations:** ^1^Department of Psychology, Peking UniversityBeijing, China; ^2^Department of Psychology, The Chinese University of Hong KongHong Kong SAR, China; ^3^Institute of Affective and Social Neuroscience, Shenzhen UniversityNanshan District, Shenzhen, Guangdong, China; ^4^Department of Psychology, Tsinghua UniversityBeijing, China

**Keywords:** aging, fall prevention, physical heath, neuropsychological factors

## Abstract

Falls, a common cause of injury among older adults, have become increasingly prevalent. As the world’s population ages, the increase in—and the prevalence of—falls among older people makes this a serious and compelling societal and healthcare issue. Physical weakness is a critical predictor in falling. While considerable research has examined this relationship, comprehensive reviews of neuropsychological predictors of falls have been lacking. In this paper, we examine and discuss current studies of the neuropsychological predictors of falls in older adults, as related to sporting and non-sporting contexts. By integrating the existing evidence, we propose that brain aging is an important precursor of the increased risk of falls in older adults. Brain aging disrupts the neural integrity of motor outputs and reduces neuropsychological abilities. Older adults may shift from unconscious movement control to more conscious or attentive motor control. Increased understanding of the causes of falls will afford opportunities to reduce their incidence, reduce consequent injuries, improve overall well-being and quality of life, and possibly to prolong life.

## Introduction

The average age of the world’s population is increasing. This rate of change is expected to accelerate in the coming decades (Lutz et al., 2008). As the number of old people throughout the world increases, senescence-related issues become increasingly important. Injury, a physical symptom degrading one’s health and quality of life, has attracted considerable attention from researchers of a variety of disciplines. A majority of studies focus on injury’s impacts on physical or physiological variables, while there is relatively scant inquiry into the neuropsychological profiles of individuals who are likely to incur a particular type of injury.

Falling is a common cause of serious injuries in community-dwelling older adults (Muir et al., 2012). In the US, the age-standardized number of years of life lost because of falls is increasing (Murray et al., 2013), making it an important topic of study in gerontology and geriatrics. Over the past few decades, a number of risk factors of falling have been identified (e.g., mobility, mental status, vision, hearing, blood pressure, hip weakness, medications, and balance control; Tinetti et al., 1986; Robbins et al., 1989; Shumway-Cook et al., 1997; Vellas et al., 1997). In addition, sarcopenia, frailty, loss of bone density, and muscle strength are well-documented and significant indicators of falls (Mühlberg and Sieber, 2004). Prospective study also finds previous fall experience as a significant predictor of future falls (Nevitt et al., 1991). In this review, evidences about the neuropsychological factors of falling in older adults are summarized and discussed, in the hope of discovering the neuropsychological characteristics of those who are most likely to fall. With this knowledge, we will have a clearer perspective for devising plans to protect those at high risk, to help improve the quality of life of the elderly, and to reduce burdens to the healthcare system.

It is estimated that 30–40% of older adults experience at least 1 fall a year. This amounts to direct costs of 0.1% and 1.5%, respectively, of the total healthcare expenditures of the United States and European countries (Ambrose et al., 2013). Most falls occur during sports or exercise, which is followed by falls while walking. It is reported that older adults are 90% more likely than their younger counterparts to fall during walking (Mertz et al., 2010).

Gait and cognition are interrelated in older adults (Montero-Odasso et al., 2012). Specifically, declines in attention, psychomotor processing, problem-solving and spatial awareness may have significant impacts on balance control and falls (Alexander and Hausdorff, 2008). For example, exposure to simultaneously high physical and cognitive demands is deleterious to balance control (Qu, 2010). Retrospective results show that people with sensory or cognitive deficits were twice as likely to fall in the past (Gauchard et al., 2006). These studies suggest that physical and neuropsychological deteriorations increase the incidence of falls in older adults (Martin et al., 2013a). The use of neuropsychological characteristics of older adults as a tool to predict the subsequent occurrence of falls bears practical importance; its reliability is, however, not well understood.

Besides neurocognitive factors, depression also has a pronounced role in predicting falling. According to the World Health Organization (2013), “depression is a common mental disorder, characterized by sadness, loss of interest or pleasure, feelings of guilt or low self-worth, disturbed sleep or appetite, feelings of tiredness and poor concentration”. Depression correlates with the incidence of falls (Delbaere et al., 2010; Sai et al., 2010). People with the most serious depressive symptoms demonstrate the highest rate of incident fall in the follow-up assessment (Eggermont et al., 2012). Prospectively, depression is related to a higher chance of recurrent falls, with an odds ratio ranging from 1.32 to 2.2 (Stalenhoef et al., 2002; Kron et al., 2003; Kerse et al., 2008; Ku et al., 2012). It is also associated with 40% and 185% increases in distal arm fracture and hip fracture risks, respectively, which are common injuries associated with falling (Kelsey et al., 2005; Hwang et al., 2011). Research shows that the connection between depression and falling is strong. Falling and depression may share a common set of risk factors (poor self-rated health, poor cognitive status, impaired functional competence, two or more visits to clinics in the past month, and a slow walking speed) (Biderman et al., 2002). However, the underlying reasons or mechanisms behind their interrelationship are still an open question.

Therefore, the main focus of this article is the neurocognitive factors of falling. We firstly examine and describe brain aging and neuropsychological decline as related to a heightened fall risk. This is followed by discussions of various neuropsychological factors, specifically global cognition, attention, executive functions, processing speed, to see how they are related to the risk of falling in older adults. A framework representing the effects of brain aging on the increased risk of falling is outlined. Finally, future research directions are presented before the summary.

## Brain aging and neuropsychological declines

Human brains undergo dramatic structural and functional changes in the course of development. While approaching old age, the cerebral volume decreases by 0.23% a year (Coffey et al., 1992). In particular, shrinkages of the frontal and pre-frontal lobes are the most significant (Coffey et al., 1992; Raz and Rodrigue, 2006; Greenwood, 2007). Consequently, when a person reaches old age, there is a greater chance that the cognitive functions supported by the frontal regions deteriorate (e.g., attention, inhibitory control). In aging, one becomes vulnerable to cognitive disorders, such as mild cognitive impairments (MCI), a transitional stage between normal aging and Alzheimer’s disease (AD). During this stage, a person experiences memory loss to an extent greater than what is expected for a given age (Smith et al., 1996; Petersen et al., 2001). It is estimated that 3% of the population have MCI (DeCarli, 2003). Although it does not take up a large proportion in the population, it deserves our attention given that MCI patients will probably soon have AD (Morris et al., 2001). Structural brain differences have been observed between people with MCI and those without. In cross-sectional studies, people with MCI show lower white matter integrity in the posterior regions and gray matter reductions in the medial temporal lobe, the insula, and the thalamus, compared to healthy controls (Karas et al., 2004; Medina et al., 2006). MCI is not only associated with cognitive dysfunctions, but also increased risks of falling (Liu-Ambrose et al., 2008b). However, it is still an open question whether the progression to cognitive disorders leads to a heightened risk of falling.

Even in healthy older adults, there is significant loss in gray and white matter, especially in the frontal and parietal lobes (Resnick et al., 2003). Loss of white matter integrity contributes to poor performance in a wide range of cognitive domains in both global and specific abilities (Longstreth et al., 1996; Gunning-Dixon et al., 2009). Degradation of white matter integrity in anterior areas is associated with reduced processing speed and working memory, while inhibitory control and task-switching are debilitated with declines in posterior areas (Kennedy and Raz, 2009). Reduced integrity in the fronto-parietal white matter contributes to increases in task-switching costs (Gold et al., 2010). Moreover, reduced frontal gray matter is associated with attention and executive function deficits (Zimmerman et al., 2006). In terms of functional changes, older adults tend to have task-specific areas over-activated, relative to young adults, for similar performance, especially in tasks requiring executive functions (Berlingeri et al., 2010; Spreng et al., 2010).

**Figure 1:**
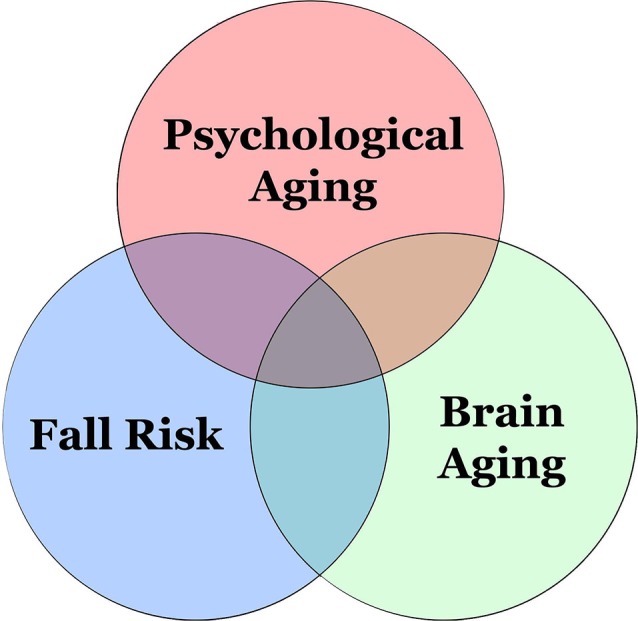
**Interrelations among fall risk and brain and psychological aging**.

Due to a shift from unconscious to increasingly conscious information-processing in older adults, reliance on frontal functions becomes greater, reflected by an increase in their pre-frontal processing (Leshikar et al., 2010). Cognitive aging comes with brain changes during the aging process. This can negatively affect motor performance and learning in older adults (Ren et al., 2013). As a person ages, motor automaticity is reduced; once seemingly easy and automatic tasks, such as walking, become more difficult or require greater conscious control (Fasano et al., 2012). It is thought that increased injury risk in older adults can be partly explained by brain and cognitive aging, in addition to frailty (Rosso et al., 2013; Figure 1). Recent evidence shows that greater white matter hyperintensity can predict falls over the following 12 months. This lends support to the existence of neural correlates of falls in older adults (Zheng et al., 2012). Identification of these neural correlates is beneficial to screen out those who are at a higher risk of falling in their older adulthood.

In addition to cognition, brain aging is related to affective disorders (e.g., depression). In depressed patients, gray and white matter integrity is reduced in the frontal areas (Hayakawa et al., 2013). White matter pathology is observed in the pre-frontal region in depressed patients (Tham et al., 2011). The disruption of frontal functions is prevalent in older adults because the frontal regions are more vulnerable in aging. Late-life depression is related to a reduction of white matter integrity in frontal-subcortical and limbic networks, which are important brain areas for emotional functions (Sexton et al., 2012). Hence, the accelerated reduction in frontal areas and related networks may contribute to the development of depression in older adults. This, in turn, leads to a heightened risk of falling.

Taken overall, deteriorations in the frontal regions and declines in frontal functions can greatly contribute to the increased risk of falling in older adults, who have a greater reliance on conscious motor control due to brain aging. Brain aging is strongly associated with (or is even a predictor of) an increased incidence of falling.

## Neuropsychological factors in falls

### Global cognition

Loss of global cognitive ability is cognitive impairment. Falls of all types are more likely to occur in older adults with cognitive impairments, as has been observed in a cross-sectional study (Hong et al., 2010). The Mini Mental State Examination (MMSE; Folstein et al., 1975) is one of the most common assessment tools for global cognition. In a prospective study, the rate of falls increases with each unit decrease in the MMSE score, and such an increase in fall incidence is evident down to an MMSE score of 22 out of 30 (Gleason et al., 2009). Another prospective study showed that older adults with poorer global cognition and executive functions are associated with a greater decline in gait speed over the following 3 years, which is modulated by depression (Atkinson et al., 2007). Furthermore, older adults with MMSE scores greater than 19 in baseline assessment experience fewer falls after multifactorial interventions designed to reduce their incidence, but such a benefit is not observed in those with low MMSE scores (Jensen et al., 2003). Thus, global cognition is associated with the risk of future falling, and it is indicative of an individual’s responsiveness to fall-reduction interventions.

Prospective results demonstrate that cognitive status can predict falls and related injuries. Cognitive status predicts the occurrence of fractures within the 2 year study period (Wilson et al., 2006). Compared with those with fractures from falling, the cognitive functions are 3.5% lower for those with any kind of fracture and 5.5% for those with a hip fracture specifically (Nordström et al., 2012). Cognitive impairment is a significant predictor of future fractures, especially in individuals with a low body mass index, which is probably due to frailty (Alfaro-Acha et al., 2006). Compared with those cognitively intact older adults, cognitively impaired older adults have a 120% greater chance of incurring fracture during a 6- to 10-year period following cognitive status assessment (Colón-Emeric et al., 2002). These same older adults also have an elevated risk of hip fractures ranging from 25% to 40% (Colón-Emeric et al., 2003; Taylor et al., 2004; Stolee et al., 2009).

### Attention

Attention is the ability to allocate cognitive resources to process the information in one’s focus, while suppressing interference and distraction. Attention span is reduced with advancing age, such that older adults can only attend to a smaller amount of information, when compared with younger people, as has been observed in cross-sectional studies (Basak and Verhaeghen, 2003; Störmer et al., 2011).

An increasing body of evidence suggests that the reduction of capacity of concentration contributes to gait and mobility disturbances (Carlson et al., 1999; Yogev-Seligmann et al., 2008; Amboni et al., 2013). The ability to concentrate is a significant predictor of gait velocity, suggesting that concentration and gait may share similar neural origins (Holtzer et al., 2006). Compared with walking alone, older adults occupied by a non-motor task while walking exhibit a reduced stride length and velocity, especially if they try to walk fast. Their natural walking pattern is disrupted by diverting their attention to a secondary task, as has been observed by de Bruin and Schmidt (2010). This situation is even more serious among balance-impaired older adults who show impairments when shifting their attention from task to task, as has been demonstrated empirically (Siu et al., 2009; Hawkes et al., 2012). Interestingly, indoor falls are mostly associated with frailty, whereas outdoor falls are linked to a lack of concentration, which may show that older adults need to exercise greater concentration in environments with which they are less familiar (Decullier et al., 2010).

Poorer performance in sustained attention was related to a greater number of falls in the coming year. Besides, mind-wandering frequency increases with number of falls (Nagamatsu et al., 2013). Additionally, poor attention performance can be associated with an increased likelihood of single and recurrent falls over the past 12 months (Holtzer et al., 2007). During aging, older adults need to make greater conscious efforts in exercising once-automatic motor skills, such as walking and balancing. Together, with a decline in their ability to concentrate, older adults can have difficulty controlling gait and balance and they have an increased risk of falling as a result.

### Executive functions

Executive functions are a set of interrelated cognitive abilities for achieving goal-directed behaviors (Banich, 2009). Inhibitory control and task-switching are the two most commonly investigated executive functions. In cross-sectional studies, older adults show greater task-switching costs (e.g., the reaction time difference in blocks requiring rule change and no rule change is greater among older adults) and poorer inhibitory control than young adults (Lindenberger et al., 2000; Cepeda et al., 2001; Germain and Collette, 2008).

Accumulating evidence has bolstered the importance of executive functions in motor control (Sheridan and Hausdorff, 2007). As is the case with attention, ample evidence suggests a close link between gait disturbances and deficits in executive functions that result from aging or sickness (Yogev-Seligmann et al., 2008). Executive functions are important both in gait control and the regulation of gait speed and variability (Amboni et al., 2013); this is because they moderate the influence of motor and sensory deficits on fall incidence prospectively (Rapport et al., 1998). They are also instrumental in coordinating multi-tasking that when confronted by dual tasks, executive functions are associated with gait and balance impairments (reduced gait speed and length and increased gait variability and body sway) (van Iersel et al., 2008; Martin et al., 2013b).

Dual-task paradigms are often used to assess executive functions. Dual-task performance is related to the incidence of falls in older adults (Beauchet et al., 2009; Hsu et al., 2012). Executive deficits can be associated with falls of any kind (Muir et al., 2012). In prospective studies, fallers are more likely to have poorer baseline executive functions than non-fallers at the follow-up, which is held 13 months after cognitive assessment (Buracchio et al., 2011). It is also evident that, in healthy older adults, those with executive functions in the lowest quartile are three times as likely to fall, at a 2 year follow-up (Herman et al., 2010).

In a longer-term prospective study, older adults with the poorest executive functions were more likely to fall sooner and to experience multiple falls (Mirelman et al., 2012). The reduced executive functions of older adults lower their judgment in motor planning, balance confidence, and decision-making while walking (Liu-Ambrose et al., 2008a, 2009; Sparto et al., 2013). Furthermore, brain activation for inhibitory control at baseline is associated with fall risk over 12-month period (Nagamatsu et al., 2011). These results show that executive functions have important impacts on the risk of falling, which may have certain neural bases. Activation and connectivity of the neural network for executive functions (e.g., frontal regions) may be able to predict future fall risks.

### Processing speed

Mental processing speed is the time taken to handle incoming information and generate behavioral outputs. Prospective results show that the risk of a non-syncopal fall (e.g., not due to a loss of consciousness, resulting in minor injuries) is increased in persons with a slower manual reaction time (Nevitt et al., 1991). Physical disability (the reduced ability to perform gross motor functions), which is a major risk factor in falls, can be explained by variance in processing speed (Binder et al., 1999). Older adults with gait disturbances have slower processing speed and poorer mood than those without (Rosano et al., 2012a). Processing speed can be a stronger predictor of falls and recurrent falls than other cognitive factors (Chen et al., 2012). Although there is preliminary support for the idea that reduced mental processing speed contributes to fall risks, given the limited available results, further studies into this cognitive component are warranted in order to reach reliable conclusions.

## An integrated framework

**Figure 2:**
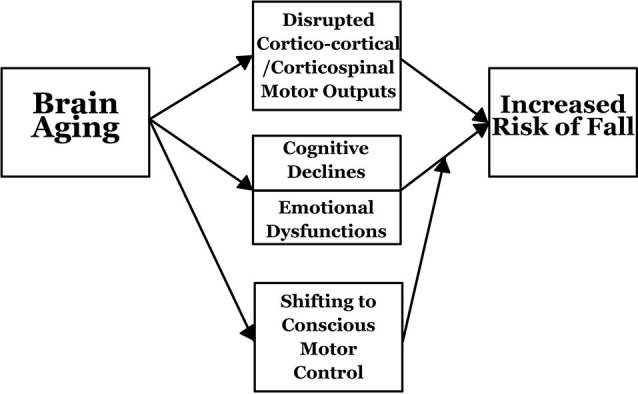
**An integrated framework of how brain aging increases risks of falling**.

The risk of falling is closely related to the aging brain and associated neuropsychological declines (Figure 2). During aging, significant structural, as well as functional, changes occur in the brain. Structural changes include reductions in cortical gray and white matter and shrinkage of brain regions (Coffey et al., 1992; Raz and Rodrigue, 2006; Greenwood, 2007). Slower gait and poorer balance control in older adults are associated with smaller gray matter volumes in regions critical for motor control (Rosano et al., 2007a). Smaller sensorimotor and fronto-parietal regions are related to shorter steps and longer double support times (Rosano et al., 2008). In addition, greater white matter disease and subclinical strokes are associated with poorer gait speed, shorter strides and longer double support time in the elderly (Rosano et al., 2006). Gray matter volume reduction of the medial temporal area and white matter hyperintensity volume are associated with gait disturbances (Rosano et al., 2012a).

Moreover, gait variability is associated with falls. It is observed that greater step length variability is related to greater subclinical brain vascular abnormalities (infarcts and white matter hyperintensities) (Rosano et al., 2007b). Longitudinal results indicate that older adults with moderate to severe brain abnormalities (ventricular enlargement, white matter hyperintensities, subcortical and basal ganglia small brain infarcts) have faster gait speed reduction than those without (Rosano et al., 2005). Functionally, compared with young adults, older people have more diffuse brain activations for the same task, probably reflecting a recruitment of additional neural resources to maintain a comparable performance to that of young people in response to cognitive declines (Ward, 2006; Park and Reuter-Lorenz, 2009; Berlingeri et al., 2010; Spreng et al., 2010).

The aging brain contributes to poorer motor performance. In older adults, compensatory phenomena are also observed for motor behaviors, showing that greater mental efforts are necessary to maintain motor performance (Ward, 2006). In addition, a loss of white matter integrity affects cortico-cortical and cortico-spinal connectivity that is possibly responsible for the reduced effectiveness of motor output and control (Madden et al., 2004). Further, brain aging can also indirectly influence fall risk through neuropsychological variables. Cognition and other neuropsychological states (e.g., emotion) are largely supported by the brain. As the brain undergoes dramatic changes while approaching older adulthood, cognitive and psychological states, such as executive functions and emotions, are adversely affected. The smaller volume of the pre-frontal area in elderly people may bring about slower gait through reducing processing speed (Rosano et al., 2012b). In young adults, routine activities, like walking and balancing, rely on subcortical control (subconscious motor control).

However, when one ages, there is a shift from unconscious to more conscious motor control that relies heavily on neuropsychological abilities. It is observed that the reduction in walking speed while working on a visual-spatial decision task is associated with 34% to 42% greater odds of recurrent falls history in the past 12 months, as has been observed in a cross-sectional study (Faulkner et al., 2007). Combined with deteriorating neuropsychological abilities, older adults’ motor control becomes more difficult, increasing their incidence of falling.

## Summary and future research

Falling is a common cause of injury in older adults and it reduces their health and overall quality of life. This review has summarized and discussed key neuropsychological factors related to falling. The identification of these factors is important in helping policy-makers, practitioners, and the older adults themselves to identify who is at risk, so that appropriate measures can be taken to avoid falling, which is crucial for ameliorating burdens to the healthcare system. Most studies have focused on the physical risk factors of falls, such as frailty; however, studies assessing the neuropsychological dimensions of falling are becoming more common. Compared to traditional questionnaire-based methods to assess fall risks, information provided by the neuropsychological approach is more objective and can be used for identifying the neurological disorders associated with falling. Although the connections between neuropsychology and falls are becoming clearer, more research on this topic is warranted. The integrated framework outlined in this review can be an impetus toward this.

The risk of falling is closely related to neuropsychological declines, especially those related to cognitive ability. Recent work has generated promising results concerning cognitive training in the elderly population. In a randomized control trial, older adults with cognitive training have their cognitive abilities improved, and the benefits persist even 5 years after cessation of the intervention (Willis et al., 2006). After training for processing speed, older adults have exhibited enhanced everyday functions, which are crucial for their independent living (Edwards et al., 2005). Computerized training has also been found to be beneficial for older adults. Training with real-time strategy video games can attenuate declines across a range of cognitive abilities like executive function and reasoning (Basak et al., 2008). Other studies have yielded positive cognitive (and some motor) outcomes after cognitive training regimens in older adults (e.g., Baltes et al., 1989; Yágüez et al., 1998; Mahncke et al., 2006). Investigating various kinds of cognitive training and the effects on fall reduction in older adults would be of great practical implication to individuals, communities, and healthcare systems worldwide.

Many of the cognitive improvements demonstrated in research have a neural basis. For example, increased brain activity is observed in the frontal and parietal cortices after working memory training (Klingberg et al., 2002). Aging is closely related to falling. A deeper understanding of the related neural mechanisms gives us better insights into the causes of falling, while providing scientists with enhanced knowledge of the neural underpinnings of the relationships between aging, cognition, and motor control. Because falling is a cause of injury involving deficiencies in motor and neuropsychological abilities, it would be reasonable that fall risks can be related by multiple neural networks, such as the frontal networks for attention, executive functions and motor planning, and the cortico-limbic emotions network.

Although a set of neuropsychological factors related to the risk of falling have been identified, their relationships and interactions are largely unknown. A complete understanding in this area will certainly help researchers to compile a more comprehensive neuropsychological profile of individuals at high risk of falling. In addition, past reports have usually investigated physical and neuropsychological factors separately. As mentioned earlier, both physical and neuropsychological factors can have neural origins related to senescence. Examining the interactions between these factors is warranted in future research.

In summary, this review has described and discussed fall risks from a neuropsychological perspective. An integrated framework that addresses brain aging and the increased risk of falls has also been put forward. This model may provide insights into the neuropsychological bases or mechanisms of falls with the hope of identifying older adults at high risk while using physical activity (e.g., Tai Chi; Yan, 1998) and cognitive regimens (e.g., video game training) to reduce the incidence of falls.

## Conflict of interest statement

The authors declare that the research was conducted in the absence of any commercial or financial relationships that could be construed as a potential conflict of interest.

## References

[B1] Alexander N. B. Hausdorff J. M. (2008). Linking thinking, walking, and falling. J. Gerontol. A Biol. Sci. Med. Sci 63, 1325–1328 10.1093/gerona/63.12.1325 19126844

[B2] Alfaro-Acha A. Ostir G. V. Markides K. S. Ottenbacher K. J. (2006). Cognitive status, body mass index, and hip fracture in older Hispanic adults. J. Am. Geriatr. Soc. 54, 1251–1255 10.1111/j.1532-5415.2006.00820.x 16913994 PMC1868446

[B3] Amboni M. Barone P. Hausdorff J. M. (2013). Cognitive contributions to gait and falls: evidence and implications. Mov. Disord. 28, 1520–1533 10.1002/mds.25674 24132840 PMC4119872

[B4] Ambrose A. F. Paul G. Hausdorff J. (2013). Risk factors for falls among older adults: a review of the literature. Maturitas 75, 51–61 10.1016/j.maturitas.2013.02.009 23523272

[B5] Atkinson H. H. Rosano C. Simonsick E. M. Williamson J. D. Davis C. Ambrosius W. T. (2007). Cognitive function, gait speed decline, and comorbidities: the health, aging and body composition study. J. Gerontol. A Biol. Sci. Med. Sci. 62, 844–850 10.1093/gerona/62.8.844 17702875

[B6] Baltes P. B. Sowarka D. Kliegl R. (1989). Cognitive training research on fluid intelligence in old age: what can older adults achieve by themselves? Psychol. Aging 4, 217–221 10.1037/0882-7974.4.2.217 2789749

[B7] Banich M. T. (2009). Executive function: the search for an integrated account. Curr. Dir. Psychol. Sci. 18, 89–94 10.1111/j.1467-8721.2009.01615.x

[B8] Basak C. Verhaeghen P. (2003). Subitizing speed, subitizing range, counting speed, the Stroop effect and aging: capacity differences and speed equivalence. Psychol. Aging 18, 240–249 10.1037/0882-7974.18.2.240 12825774

[B9] Basak C. Boot W. R. Voss M. W. Kramer A. F. (2008). Can training in a real-time strategy video game attenuate cognitive decline in older adults? Psychol. Aging 23, 765–777 10.1037/a0013494 19140648 PMC4041116

[B10] Beauchet O. Annweiler C. Dubost V. Allali G. Kressig R. W. Bridenbaugh S. (2009). Stops walking when talking: a predictor of falls in older adults? Eur. J. Neurol. 16, 786–795 10.1111/j.1468-1331.2009.02612.x 19473368

[B11] Berlingeri M. Bottini G. Danelli L. Ferri F. Traficante D. Sacheli L. (2010). With time on our side? Task-dependent compensatory processes in graceful aging. Exp. Brain Res. 205, 307–324 10.1007/s00221-010-2363-7 20680252

[B12] Biderman A. Cwikel J. Fried A. V. Galinsky D. (2002). Depression and falls among community dwelling elderly people: a search for common risk factors. J. Epidemiol. Community Health 56, 631–636 10.1136/jech.56.8.631 12118057 PMC1732215

[B13] Binder E. F. Storandt M. Birge S. J. (1999). The relation between psychometric test performance and physical performance in older adults. J. Gerontol. A Biol. Sci. Med. Sci. 54, M428–M432 10.1093/gerona/54.8.m428 10496549

[B14] Buracchio T. J. Mattek N. C. Dodge H. H. Hayes T. L. Pavel M. Howieson D. B. (2011). Executive function predicts risk of falls in older adults without balance impairment. BMC Geriatr. 11:74 10.1186/1471-2318-11-74 22070602 PMC3226437

[B15] Carlson M. C. Fried L. P. Xue Q. L. Bandeen-Roche K. Zeger S. L. Brandt J. (1999). Association between executive attention and physical functional performance in community-dwelling older women. J. Gerontol. B Psychol. Sci. Soc. Sci. 54, S262–S270 10.1093/geronb/54b.5.s262 10542828

[B16] Cepeda N. J. Kramer A. F. Gonzalez de Sather J. C. M. (2001). Changes in executive control across the life span: examination of task-switching performance. Dev. Psychol. 37, 715–730 10.1037//0012-1649.37.5.715 11552766

[B17] Chen T. Y. Peronto C. L. Edwards J. D. (2012). Cognitive function as a prospective predictor of falls. J. Gerontol. B Psychol. Sci. Soc. Sci. 67, 720–728 10.1093/geronb/gbs052 22865822 PMC3636670

[B18] Coffey C. E. Wilkinson W. E. Parashos L. A. Soady S. A. R. Sullivan R. J. Patterson L. J. (1992). Quantitative cerebral anatomy of the aging human brain: a cross-sectional study using magnetic resonance imaging. Neurology 42, 527–536 10.1212/wnl.42.3.527 1549213

[B19] Colón-Emeric C. S. Biggs D. P. Schenck A. P. Lyles K. W. (2003). Risk factors for hip fracture in skilled nursing facilities: who should be evaluated? Osteoporos. Int. 14, 484–489 10.1007/s00198-003-1384-5 12730734

[B20] Colón-Emeric C. S. Pieper C. F. Artz M. B. (2002). Can historical and functional risk factors be used to predict fractures in community-dwelling older adults? Development and validation of a clinical tool. Osteoporos. Int. 13, 955–961 10.1007/s001980200133 12459938

[B21] de Bruin E. D. Schmidt A. (2010). Walking behavior of healthy elderly: attention should be paid. Behav. Brain Funct. 6:59 10.1186/1744-9081-6-59 20939911 PMC2959004

[B22] DeCarli C. (2003). Mild cognitive impairment: prevalence, prognosis, aetiology, and treatment. Lancet Neurol. 2, 15–21 10.1016/s1474-4422(03)00262-x 12849297

[B23] Decullier E. Couris C. M. Beauchet O. Zamora A. Annweiler C. Dargent-Molina P. (2010). Falls’ and fallers’ profiles. J. Nutr. Health Aging 14, 602–608 10.1007/s12603-010-0130-x 20818477 PMC12880277

[B24] Delbaere K. Close J. C. Heim J. Sachdev P. S. Brodaty H. Slavin M. J. (2010). A multifactorial approach to understanding fall risk in older people. J. Am. Geriatr. Soc. 58, 1679–1685 10.1111/j.1532-5415.2010.03017.x 20863327

[B25] Edwards J. D. Wadley V. G. Vance D. E. Wood K. Roenker D. L. Ball K. K. (2005). The impact of speed of processing training on cognitive and everyday performance. Aging Ment. Health 9, 262–271 10.1080/13607860412331336788 16019280

[B26] Eggermont L. H. Penninx B. W. Jones R. N. Leveille S. G. (2012). Depressive symptoms, chronic pain, and falls in older community-dwelling adults: the MOBILIZE Boston Study. J. Am. Geriatr. Soc. 60, 230–237 10.1111/j.1532-5415.2011.03829.x 22283141 PMC3288166

[B27] Fasano A. Plotnik M. Bove F. Berardelli A. (2012). The neurobiology of falls. Neurol. Sci. 33, 1215–1223 10.1007/s10072-012-1126-6 22673818

[B28] Faulkner K. A. Redfern M. S. Cauley J. A. Landsittel D. P. Studenski S. A. Rosano C. (2007). Multitasking: association between poorer performance and a history of recurrent falls. J. Am. Geriatr. Soc. 55, 570–576 10.1111/j.1532-5415.2007.01147.x 17397436

[B29] Folstein M. F. Folstein S. E. McHugh P. R. (1975). ‘Mini-mental state’. A practical method for grading the cognitive state of patients for the clinician. J. Psychiatr. Res. 12, 189–198 10.1016/0022-3956(75)90026-6 1202204

[B30] Gauchard G. C. Deviterne D. Guillemin F. Sanchez J. Perrin P. P. Mur J. M. (2006). Prevalence of sensory and cognitive disabilities and falls and their relationships: a community-based study. Neuroepidemiology 26, 108–118 10.1159/000090445 16374036

[B31] Germain S. Collette F. (2008). Dissociation of perceptual and motor inhibitory processes in young and elderly participants using the Simon task. J. Int. Neuropsychol. Soc. 14, 1014–1021 10.1017/s135561770808123x 18954481

[B32] Gleason C. E. Gangnon R. E. Fischer B. L. Mahoney J. E. (2009). Increased risk for falling associated with subtle cognitive impairment: secondary analysis of a randomized clinical trial. Dement. Geriatr. Cogn. Disord. 27, 557–563 10.1159/000228257 19602883 PMC2742559

[B33] Gold B. T. Powell D. K. Xuan L. Jicha G. A. Smith C. D. (2010). Age-related slowing of task switching is associated with decreased integrity of frontoparietal white matter. Neurobiol. Aging 31, 512–522 10.1016/j.neurobiolaging.2008.04.005 18495298 PMC2815097

[B34] Greenwood P. M. (2007). Functional plasticity in cognitive aging: review and hypothesis. Neuropsychology 21, 657–673 10.1037/0894-4105.21.6.657 17983277

[B35] Gunning-Dixon F. M. Brickman A. M. Cheng J. C. Alexopoulos G. S. (2009). Aging of cerebral white matter: a review of MRI findings. Int. J. Geriatr. Psychiatry 24, 109–117 10.1002/gps.2087 18637641 PMC2631089

[B36] Hawkes T. D. Siu K. C. Silsupadol P. Woollacott M. H. (2012). Why does older adults’ balance become less stable when walking and performing a secondary task? Examination of attentional switching abilities. Gait Posture 35, 159–163 10.1016/j.gaitpost.2011.09.001 21964051 PMC3251721

[B37] Hayakawa Y. K. Sasaki H. Takao H. Mori H. Hayashi N. Kunimatsu A. (2013). Structural brain abnormalities in women with subclinical depression, as revealed by voxel-based morphometry and diffusion tensor imaging. J. Affect. Disord. 144, 263–268 10.1016/j.jad.2012.10.023 23141669

[B38] Herman T. Mirelman A. Giladi N. Schwiger A. Hausforff J. M. (2010). Executive control deficits as a prodrome to falls in healthy older adults: a prospective study linking thinking, walking, and falling. J. Gerontol. A Biol. Sci. Med. Sci. 65, 1086–1092 10.1093/gerona/glq077 20484336 PMC2949331

[B39] Holtzer R. Friedman R. Lipton R. B. Katz M. Xue X. Verghese J. (2007). The relationship between specific cognitive functions and falls in aging. Neuropsychology 21, 540–548 10.1037/0894-4105.21.5.540 17784802 PMC3476056

[B40] Holtzer R. Verghese J. Xue X. Lipton R. B. (2006). Cognitive processes related to gait velocity: results from the Einstein Aging Study. Neuropsychology 20, 215–223 10.1037/0894-4105.20.2.215 16594782

[B41] Hong G. R. S. Cho S. H. Tak Y. (2010). Falls among Koreans 45 years of age and older: incidence and risk factors. J. Adv. Nurs. 66, 2014–2024 10.1111/j.1365-2648.2010.05384.x 20626472

[B42] Hsu C. L. Nagamatsu L. S. Davis J. C. Liu-Ambrose T. (2012). Examining the relationship between specific cognitive processes and falls risk in older adults: a systematic review. Osteoporos. Int. 23, 2409–2424 10.1007/s00198-012-1992-z 22638707 PMC4476839

[B43] Hwang H. F. Lee H. D. Huang H. H. Chen C. Y. Lin M. R. (2011). Fall mechanisms, bone strength, and hip fractures in elderly men and women in Taiwan. Osteoporos. Int. 22, 2385–2393 10.1007/s00198-010-1446-4 20963399

[B44] Jensen J. Nyberg L. Gustafson Y. Lundin-Olsson L. (2003). Fall and injury prevention in residential care – effects in residents with higher and lower levels of cognition. J. Am. Geriatr. Soc. 51, 627–635 10.1034/j.1600-0579.2003.00206.x 12752837

[B45] Karas G. B. Scheltens P. Rombouts S. A. R. B. Visser P. J. Van Schijndel R. A. Fox N. C. (2004). Global and local gray matter loss in mild cognitive impairment and Alzheimer’s disease. Neuroimage 23, 708–716 10.1016/j.neuroimage.2004.07.006 15488420

[B46] Kelsey J. L. Prill M. M. Keegan T. H. Tanner H. E. Bernstein A. L. Quesenberry C. P. Jr. (2005). Reducing the risk for distal forearm fracture: preserve bone mass, slow down and don’t fall! Osteoporos. Int. 16, 681–690 10.1007/s00198-004-1745-8 15517189

[B47] Kennedy K. M. Raz N. (2009). Aging white matter and cognition: differential effects of regional variations in diffusion properties on memory, executive functions, and speed. Neuropsychologia 47, 916–927 10.1016/j.neuropsychologia.2009.01.001 19166865 PMC2643310

[B48] Kerse N. Flicker L. Pfaff J. J. Draper B. Lautenschlager N. T. Sim M. (2008). Falls, depression and antidepressants in later life: a large primary care appraisal. PLoS One 3:e2423 10.1371/journal.pone.0002423 18560599 PMC2413407

[B49] Klingberg T. Forssberg H. Westerberg H. (2002). Increased brain activity in frontal and parietal cortex underlies the development of visuospatial working memory capacity during childhood. J. Cogn. Neurosci. 14, 1–10 10.1162/089892902317205276 11798382

[B50] Kron M. Loy S. Sturm E. Nikolaus T. H. Becker C. (2003). Risk indicators for falls in institutionalized frail elderly. Am. J. Epidemiol. 158, 645–653 10.1093/aje/kwg203 14507600

[B51] Ku Y. C. Liu M. E. Tsai Y. F. Liu W. C. Lin S. L. Tsai S. J. (2012). Associated factors for falls, recurrent falls, and injurious falls in aged men living in Taiwan veterans homes. Int. J. Gerontol. 7, 80–84 10.1016/j.ijge.2012.07.004

[B52] Leshikar E. D. Gutchess A. H. Hebrank A. C. Sutton B. P. Park D. C. (2010). The impact of increased relational encoding demands on frontal and hippocampal function in older adults. Cortex 46, 507–521 10.1016/j.cortex.2009.07.011 19709652 PMC2826535

[B53] Lindenberger U. Marsiske M. Baltes P. B. (2000). Memorizing while walking: increase in dual-task costs from young adulthood to old age. Psychol. Aging 15, 417–436 10.1037/0882-7974.15.3.417 11014706

[B54] Liu-Ambrose T. Ahamed Y. Graf P. Feldman F. Robinovitch S. N. (2008a). Older fallers with poor working memory overestimate their postural limits. Arch. Phys. Med. Rehabil. 89, 1335–1340 10.1016/j.apmr.2007.11.052 18586136

[B55] Liu-Ambrose T. Y. Ashe M. C. Graf P. Beattie B. L. Khan K. M. (2008b). Increased risk of falling in older community-dwelling women with mild cognitive impairment. Phys. Ther. 88, 1482–1491 10.2522/ptj.20080117 18820094 PMC3514550

[B56] Liu-Ambrose T. Katarynych L. A. Ashe M. C. Nagamatsu L. S. Hsu C. L. (2009). Dual-task gait performance among community-dwelling senior women: the role of balance confidence and executive functions. J. Gerontol. A Biol. Sci. Med. Sci. 64, 975–982 10.1093/gerona/glp063 19429702

[B57] Longstreth W. T. Manolio T. A. Arnold A. Burke G. L. Bryan N. Jungreis C. A. (1996). Clinical correlates of white matter findings on cranial magnetic resonance imaging of 3301 elderly people: the Cardiovascular Health Study. Stroke 27, 1274–1282 10.1161/01.str.27.8.1274 8711786

[B58] Lutz W. Sanderson W. Scherbov S. (2008). The coming acceleration of global population ageing. Nature 451, 716–719 10.1038/nature06516 18204438

[B59] Madden D. J. Whiting W. L. Huettel S. A. White L. E. MacFall J. R. Provenzale J. M. (2004). Diffusion tensor imaging of adult age differences in cerebral white matter: relation to response time. Neuroimage 21, 1174–1181 10.1016/j.neuroimage.2003.11.004 15006684

[B60] Mahncke H. W. Connor B. B. Appelman J. Ahsanuddin O. N. Hardy J. L. Wood R. A. (2006). Memory enhancement in healthy older adults using a brain plasticity-based training program: a randomized, controlled study. Proc. Natl. Acad. Sci. U S A 103, 12523–12528 10.1073/pnas.0605194103 16888038 PMC1526649

[B61] Martin K. L. Blizzard L. Srikanth V. K. Wood A. Thomson R. Sanders L. M. (2013a). Cognitive function modifies the effect of physiological function on the risk of multiple falls—a population-based study. J. Gerontol. A Biol. Sci. Med. Sci. 68, 1091–1097 10.1093/gerona/glt010 23410920

[B62] Martin K. L. Blizzard L. Wood A. G. Srikanth V. Thomson R. Sanders L. M. (2013b). Cognitive function, gait, and gait variability in older people: a population-based study. J. Gerontol. A Biol. Sci. Med. Sci. 68, 726–732 10.1093/gerona/gls224 23112113

[B63] Medina D. DeToledo-Morrell L. Urresta F. Gabrieli J. D. Moseley M. Fleischman D. (2006). White matter changes in mild cognitive impairment and AD: a diffusion tensor imaging study. Neurobiol. Aging 27, 663–672 10.1016/j.neurobiolaging.2005.03.026 16005548

[B64] Mertz K. J. Lee D. C. Sui X. Powell K. E. Blair S. N. (2010). Falls among adults: the association of cardiorespiratory fitness and physical activity with walking-related falls. Am. J. Prev. Med. 39, 15–24 10.1016/j.amepre.2010.03.013 20547276 PMC2897244

[B65] Mirelman A. Herman T. Brozgol M. Dorfman M. Sprecher E. Schweiger A. (2012). Executive function and falls in older adults: new findings from a five-year prospective study link fall risk to cognition. PLoS One 7:e40297 10.1371/journal.pone.0040297 22768271 PMC3386974

[B66] Montero-Odasso M. Verghese J. Beauchet O. Hausdorff J. M. (2012). Gait and cognition: a complementary approach to understanding brain function and the risk of falling. J. Am. Geriatr. Soc. 60, 2127–2136 10.1111/j.1532-5415.2012.04209.x 23110433 PMC3498517

[B67] Morris J. C. Storandt M. Miller J. P. McKeel D. W. Price J. L. Rubin E. H. (2001). Mild cognitive impairment represents early-stage Alzheimer disease. Arch. Neurol. 58, 397–405 10.1001/archneur.58.3.397 11255443

[B68] Mühlberg W. Sieber C. (2004). Sarcopenia and frailty in geriatric patients: implications for training and prevention. Z. Gerontol. Geriatr. 37, 2–8 10.1007/s00391-004-0203-8 14991289

[B69] Muir S. W. Gopaul K. Odasso M. M. M. (2012). The role of cognitive impairment in fall risk among older adults: a systematic review and meta-analysis. Age Ageing 41, 299–308 10.1093/ageing/afs012 22374645

[B70] Murray C. J. Abraham J. Ali M. K. Alvarado M. Atkinson C. Baddour L. M. (2013). The state of US health, 1990–2010: burden of diseases, injuries and risk factors. JAMA 310, 591–608 10.1001/jama.2013.13805 23842577 PMC5436627

[B71] Nagamatsu L. S. Hsu C. L. Handy T. C. Liu-Ambrose T. (2011). Functional neural correlates of reduced physiological falls risk. Behav. Brain Funct. 7:37 10.1186/1744-9081-7-37 21846395 PMC3178476

[B72] Nagamatsu L. S. Kam J. W. Liu-Ambrose T. Chan A. Handy T. C. (2013). Mind-wandering and falls risk in older adults. Psychol. Aging 28, 685–691 10.1037/a0034197 24041001 PMC4357518

[B73] Nevitt M. C. Cummings S. R. Hudes E. S. (1991). Risk factors for injurious falls: a prospective study. J. Gerontol. 46, M164–M170 10.1093/geronj/46.5.m164 1890282

[B75] Nordström P. Franks P. W. Gustafson Y. Nordström A. (2012). Cognitive function in young men and the later risk of fractures. J. Bone Miner. Res. 27, 2291–2297 10.1002/jbmr.1683 22692934

[B76] Park D. C. Reuter-Lorenz P. (2009). The adaptive brain: aging and neurocognitive scaffolding. Annu. Rev. Psychol. 60, 173–196 10.1146/annurev.psych.59.103006.093656 19035823 PMC3359129

[B77] Petersen R. C. Doody R. Kurz A. Mohs R. C. Morris J. C. Rabins P. V. (2001). Current concepts in mild cognitive impairment. Arch. Neurol. 58, 1985–1992 10.1001/archneur.58.12.1985 11735772

[B78] Qu X. (2010). Physical load handling and listening comprehension effects on balance control. Ergonomics 53, 1461–1467 10.1080/00140139.2010.529167 21108083

[B79] Rapport L. J. Hanks R. A. Millis S. R. Deshpande S. A. (1998). Executive functioning and predictors of falls in the rehabilitation setting. Arch. Phys. Med. Rehabil. 79, 629–633 10.1016/S0003-9993(98)90035-1 9630140

[B80] Raz N. Rodrigue K. M. (2006). Differential aging of the brain: patterns, cognitive correlates and modifiers. Neurosci. Biobehav. Rev. 30, 730–748 10.1016/j.neubiorev.2006.07.001 16919333 PMC6601348

[B81] Ren J. Wu Y. D. Chan J. S. Yan J. H. (2013). Cognitive aging affects motor performance and learning. Geriatr. Gerontol. Int. 13, 19–27 10.1111/j.1447-0594.2012.00914.x 22817645

[B82] Resnick S. M. Pham D. L. Kraut M. A. Zonderman A. B. Davatzikos C. (2003). Longitudinal magnetic resonance imaging studies of older adults: a shrinking brain. J. Neurosci. 23, 3295–3301 12716936 10.1523/JNEUROSCI.23-08-03295.2003 PMC6742337

[B83] Robbins A. S. Rubenstein L. Z. Josephson K. R. Schulman B. L. Osterweil D. Fine G. (1989). Predictors of falls among elderly people: results of two population-based studies. Arch. Intern. Med. 149, 1628–1633 10.1001/archinte.149.7.1628 2742437

[B84] Rosano C. Aizenstein H. J. Studenski S. Newman A. B. (2007a). A regions-of-interest volumetric analysis of mobility limitations in community-dwelling older adults. J. Gerontol. A Biol. Sci. Med. Sci. 62, 1048–1055 10.1093/gerona/62.9.1048 17895446

[B85] Rosano C. Aizenstein H. Brach J. Longenberger A. Studenski S. Newman A. B. (2008). Gait measures indicate underlying focal gray matter atrophy in the brain of older adults. J. Gerontol. A Biol. Sci. Med. Sci. 63, 1380–1388 10.1093/gerona/63.12.1380 19126852 PMC2648808

[B86] Rosano C. Bennett D. A. Newman A. B. Venkatraman V. Yaffe K. Harris T. (2012a). Patterns of focal gray matter atrophy are associated with bradykinesia and gait disturbances in older adults. J. Gerontol. A Biol. Sci. Med. Sci. 67, 957–962 10.1093/gerona/glr262 22367436 PMC3436092

[B87] Rosano C. Brach J. Longstreth W. T. Jr. Newman A. B. (2006). Quantitative measures of gait characteristics indicate prevalence of underlying subclinical structural brain abnormalities in high-functioning older adults. Neuroepidemiology 26, 52–60 10.1159/000089240 16254454

[B88] Rosano C. Brach J. Studenski S. Longstreth W. T. Jr. Newman A. B. (2007b). Gait variability is associated with subclinical brain vascular abnormalities in high-functioning older adults. Neuroepidemiology 29, 193–200 10.1159/000111582 18043004 PMC2824582

[B89] Rosano C. Kuller L. H. Chung H. Arnold A. M. Longstreth W. T. Jr. Newman A. B. (2005). Subclinical brain magnetic resonance imaging abnormalities predict physical functional decline in high-functioning older adults. J. Am. Geriatr. Soc. 53, 649–654 10.1111/j.1532-5415.2005.53214.x 15817012

[B90] Rosano C. Studenski S. A. Aizenstein H. J. Boudreau R. M. Longstreth W. T. Jr. Newman A. B. (2012b). Slower gait, slower information processing and smaller prefrontal area in older adults. Age Ageing 41, 58–64 10.1093/ageing/afr113 21965414 PMC3234076

[B91] Rosso A. L. Studenski S. A. Chen W. G. Aizenstein H. J. Alexander N. B. Bennett D. A. (2013). Aging, the central nervous system, and mobility. J. Gerontol. A Biol. Sci. Med. Sci. 68, 1379–1386 10.1093/gerona/glt089 23843270 PMC3805295

[B92] Sai A. J. Gallagher J. C. Smith L. M. Logsdon S. (2010). Fall predictors in the community dwelling elderly: a cross sectional and prospective cohort study. J. Musculoskelet. Neuronal Interact. 10, 142–150 20516631

[B93] Sexton C. E. Allan C. L. Le Masurier M. McDermott L. M. Kalu U. G. Herrmann L. L. (2012). Magnetic resonance imaging in late-life depression multimodal examination of network disruption. Arch. Gen. Psychiatry 69, 680–689 10.1001/archgenpsychiatry.2011.1862 22752234

[B94] Sheridan P. L. Hausdorff J. M. (2007). The role of higher-level cognitive function in gait: executive dysfunction contributes to fall risk in Alzheimer’s disease. Dement. Geriatr. Cogn. Disord. 24, 125–137 10.1159/000105126 17622760 PMC3163262

[B95] Shumway-Cook A. Baldwin M. Polissar N. L. Gruber W. (1997). Predicting the probability for falls in community-dwelling older adults. Phys. Ther. 77, 812–819 9256869 10.1093/ptj/77.8.812

[B96] Siu K. C. Chou L. S. Mayr U. van Donkelaar P. Woollacott M. H. (2009). Attentional mechanisms contributing to balance constraints during gait: the effects of balance impairments. Brain Res. 1248, 59–67 10.1016/j.brainres.2008.10.078 19028462 PMC3133742

[B97] Smith G. E. Petersen R. C. Parisi J. E. Ivnik R. J. Kokmen E. Tangalos E. G. (1996). Definition, course and outcome of mild cognitive impairment. Aging Neuropsychol. Cogn. 3, 141–147 10.1080/13825589608256619

[B98] Sparto P. J. Fuhrman S. I. Redfern M. S. Jennings J. R. Perera S. Nebes R. D. (2013). Postural adjustment errors reveal deficits in inhibition during lateral step initiation in older adults. J. Neurophysiol. 109, 415–428 10.1152/jn.00682.2012 23114211 PMC3545456

[B99] Spreng R. N. Wojtowicz M. Grady C. L. (2010). Reliable differences in brain activity between young and old adults: a quantitative meta-analysis across multiple cognitive domains. Neurosci. Biobehav. Rev. 34, 1178–1194 10.1016/j.neubiorev.2010.01.009 20109489

[B100] Stalenhoef P. A. Diederiks J. P. M. Knottnerus J. A. Kester A. D. M. Crebolder H. F. J. M. (2002). A risk model for the prediction of recurrent falls in community-dwelling elderly: a prospective cohort study. J. Clin. Epidemiol. 55, 1088–1094 10.1016/s0895-4356(02)00502-4 12507672

[B101] Stolee P. Poss J. Cook R. J. Byrne K. Hirdes J. P. (2009). Risk factors for hip fracture in older home care clients. J. Gerontol. A Biol. Sci. Med. Sci. 64, 403–410 10.1093/gerona/gln035 19196903 PMC2654998

[B102] Störmer V. S. Li S. C. Heekeren H. R. Lindenberger U. (2011). Feature-based interference from unattended visual field during attentional tracking in younger and older adults. J. Vis. 11:1 10.1167/11.2.1 21285297

[B103] Taylor B. C. Schreiner P. J. Stone K. L. Fink H. A. Cummings S. R. Nevitt M. C. (2004). Long-term prediction of incident hip fracture risk in elderly white women: study of osteoporotic fractures. J. Am. Geriatr. Soc. 52, 1479–1486 10.1111/j.1532-5415.2004.52410.x 15341549

[B104] Tham M. W. Woon P. S. Sum M. Y. Lee T. S. Sim K. (2011). White matter abnormalities in major depression: evidence from post-mortem, neuroimaging and genetic studies. J. Affect. Disord. 132, 26–36 10.1016/j.jad.2010.09.013 20889213

[B105] Tinetti M. E. Williams T. F. Mayewski R. (1986). Fall risk index for elderly patients based on number of chronic disabilities. Am. J. Med. 80, 429–434 10.1016/0002-9343(86)90717-5 3953620

[B106] van Iersel M. B. Kessels R. P. Bloem B. R. Verbeek A. L. Rikkert M. G. O. (2008). Executive functions are associated with gait and balance in community-living elderly people. J. Gerontol. A Biol. Sci. Med. Sci. 63, 1344–1349 10.1093/gerona/63.12.1344 19126847

[B107] Vellas B. J. Wayne S. J. Romero L. Baumgartner R. N. Rubenstein L. Z. Garry P. J. (1997). One-leg balance is an important predictor of injurious falls in older persons. J. Am. Geriatr. Soc. 45, 735–738 9180669 10.1111/j.1532-5415.1997.tb01479.x

[B108] Ward N. S. (2006). Compensatory mechanisms in the aging motor system. Ageing Res. Rev. 5, 239–254 10.1016/j.arr.2006.04.003 16905372

[B109] Willis S. L. Tennstedt S. L. Marsiske M. Ball K. Elias J. Koepke K. M. (2006). Long-term effects of cognitive training on everyday functional outcomes in older adults. JAMA 296, 2805–2814 10.1001/jama.296.23.2805 17179457 PMC2910591

[B110] Wilson R. T. Chase G. A. Chrischilles E. A. Wallace R. B. (2006). Hip fracture risk among community-dwelling elderly people in the United States: a prospective study of physical, cognitive and socioeconomic indicators. Am. J. Public Health 96, 1210–1218 10.2105/ajph.2005.077479 16735617 PMC1483878

[B111] World Health Organization. (2013). Depression.from http://www.who.int/topics/depression/en/ Retrieved November 1st, 2013

[B112] Yágüez L. Nagel D. Hoffman H. Canavan A. G. M. Wist E. Hömberg V. (1998). A mental route to motor learning: improving trajectorial kinematics through imagery training. Behav. Brain Res. 90, 95–106 10.1016/s0166-4328(97)00087-9 9520217

[B113] Yan J. H. (1998). Tai Chi practice improves senior citizens’ balance and arm movement control. J. Aging Phys. Act. 6, 271–284

[B114] Yogev-Seligmann G. Hausdorff J. M. Giladi N. (2008). The role of executive function and attention in gait. Mov. Disord. 23, 329–342 10.1002/mds.21720 18058946 PMC2535903

[B115] Zheng J. J. Lord S. R. Close J. C. Sachdev P. S. Wen W. Brodaty H. (2012). Brain white matter hyperintensities, executive dysfunction, instability, and falls in older people: a prospective cohort study. J. Gerontol. A Biol. Sci. Med. Sci. 67, 1085–1091 10.1093/gerona/gls063 22403055

[B116] Zimmerman M. E. Brickman A. M. Paul R. H. Grieve S. M. Tate D. F. Gunstad J. (2006). The relationship between frontal gray matter volume and cognition varies across the healthy adult lifespan. Am. J. Geriatr. Psychiatry 14, 823–833 10.1097/01.JGP.0000238502.40963.ac 17001022

